# A study on predicting the risk of coronary artery disease in OSAHS patients based on a four-variable screening tool potential predictive model and its correlation with the severity of coronary atherosclerosis

**DOI:** 10.3389/fcvm.2025.1602492

**Published:** 2025-06-27

**Authors:** Yanli Yao, Yu Li, Yulan Chen, Xuan Qiu, Gulimire Aimaiti, Ayiguzaili Maimaitimin

**Affiliations:** ^1^ Department of Hypertension, The First Affiliated Hospital of Xinjiang Medical University, Urumqi, China; ^2^ Second Department of Comprehensive Internal Medicine, The First Affiliated Hospital of Xinjiang Medical University, Urumqi, China

**Keywords:** four-variable screening tool, obstructive sleep apnea hypopnea syndrome, coronary artery disease, prediction, association study

## Abstract

**Objective:**

This study aims to evaluate the potential association between the four-variable screening tool (the 4 V) potential predictive model in predicting coronary artery disease (CAD) risk in patients with obstructive sleep apnea-hypopnea syndrome (OSAHS) and its correlation with the severity of coronary atherosclerosis, as measured by the Gensini scoring system.

**Methods:**

1197 OSAHS patients with suspected CAD who were hospitalized in the First Affiliated Hospital of Xinjiang Medical University between March 2020 and February 2024 were selected. The patients were submitted to coronary angiography or Coronary Computed Tomography Angiography (CCTA) examination to confirm the diagnosis. There were 423 cases in the OSAHS plus CAD group and 774 cases in the OSAHS group. LASSO regression analysis was carried out for screening potential influencing factors. Propensity score matching (PSM) was used to balance covariables between groups, and 293 cases were included per group in a 1:1 ratio. Univariable and multivariable logistic regression analyses were employed to evaluate parameters independently associated with CAD and construct a nomogram model.Receiver operating characteristic (ROC) curve analysis, Hosmer-Lemeshow test, calibration curve and decision curve (DCA) analyses were employed to assess its predictive value in CAD. A random forest machine learning algorithm was used to evaluate the importance of each risk factor. *Pearson's or Spearman's* correlation coefficients were employed to assess the strengths of associations among all variables and between predictors and Gensini scores, reflected in heat maps and chord diagrams, respectively.

**Results:**

LASSO-logistic regression analysis revealed age (*OR* = 1.07, 95% *CI*: 1.05–1.1, *P* < 0.001), hypertension (*OR* = 1.29, 95% *CI*: 1.16–1.44, *P* < 0.001), AHI (*OR* = 1.02, 95% *CI*: 1.01–1.03, *P* = 0.007), and the 4 V (*OR* = 1.84, 95% *CI*: 1.21–2.79, *P* = 0.004) were independently associated with OSAHS plus CAD. The analysis of the ROC curve revealed that the combined utilization of the aforementioned predictors significantly enhances the potential predictive capability for patients with OSAHS developing CAD. The Hosmer-Lemeshow test, calibration curve, and DCA results indicate that potential predictive model based on the 4 V possesses significant clinical applicability in predicting OSAHS in conjunction with CAD. A comprehensive analysis utilizing the random forest machine learning algorithm demonstrated that the AHI exhibits the highest predictive value. Furthermore, the model's performance, as evaluated through out-of-bag error assessment, suggests robust efficacy. The correlation analysis results showed that the scores of the four-variable screening tool were positively correlated with the Gensini scores.

**Conclusion:**

Age, hypertension, AHI, and the four-variable screening tool are independent risk factors for CAD in patients with OSAHS. The potential predictive model based on the 4 V is closely related to the prediction of CAD and its correlation with the severity of coronary atherosclerosis.

## Introduction

Obstructive Sleep Apnea Hypopnea Syndrome (OSAHS) represents an important respiratory disease related to sleep-perturbed breathing, often accompanied by upper airway obstruction, which causes patients to experience recurrent intermittent hypoxia, thereby activating central and peripheral chemoreceptors, triggering a series of physiological reactions (1). The latest data show about 936 million individuals aged 30–69 years worldwide suffer from OSAHS, and China has the largest number of affected people, with a prevalence rate as high as 18.8% (2). OSAHS is closely related to multiple cardiovascular diseases, including CAD, hypertension, arrhythmia, heart failure, etc., which greatly decrease the patient's quality of life and life expectancy (3).

Polysomnography (PSG) is the gold standard for diagnosing OSAHS. However, it is not suitable for large-scale disease screening due to shortcomings such as the need for specialized equipment and technicians, complex operating procedures, high cost, and limitations of hospital resources. Therefore, simplified OSAHS screening scales have attracted increasing attention. These scales are efficient, convenient, and easy to applied, and mostly include the 4V (4), BQ (5), SBQ (6), etc.

Recurrent intermittent hypoxia in OSAHS can activate a series of pathophysiological processes, including oxidative stress, inflammatory response, sympathetic activation, endothelial dysfunction, etc. These pathological changes not only increase the burden on the cardiovascular system, but also promote CAD. The formation and development of atherosclerotic plaques are considered crucial factors in CAD occurrence and development (7). CAD prevalence in OSAHS patients is 20% to 30% (8), while OSAHS prevalence in CAD cases is as high as 38% to 65% (9). People with OSAHS show starkly increased risk of worsened underlying cardiovascular disease and CAD development. Studies have shown that the risk of death is higher in cases with concurrent OSAHS and CAD compared with individuals with OSAHS or CAD alone; therefore, early risk stratification and primary prevention of OSAHS patients who may develop CAD is crucial (10). The 2024 Expert Consensus on the Evaluation and Management of Obstructive Sleep Apnea in Patients With Cardiovascular Disease (11) recommends the STOP-Bang questionnaire for preliminary screening of patients with suspected OSAHS combined with Cardiovascular Disease(CVD). The SBQ encompasses three subjective variables: snoring, fatigue, and observed apnea, with results significantly influenced by subjective factors. The 4 V includes only one subjective variable, snoring, potentially enhances its efficiency in practical applications. However, the relationship between this tool and coronary artery disease in patients with OSAHS remains unclear. This study aims to evaluate the 4 V for predicting the risk of CAD in OSAHS patients and its potential association with the severity of coronary atherosclerosis.

## Methods

### Study population

A total of 1,197 OSAHS patients with suspected CAD who were hospitalized at the First Affiliated Hospital of Xinjiang Medical University between March 2020 and February 2024 were selected (Individuals who meet the following criteria can be defined as “suspected CAD”: Typical angina symptoms, or atypical symptoms with multiple risk factors, or non-invasive tests indicating myocardial ischemia (such as abnormal ECG or stress test results). All patients underwent coronary angiography or CCTA examination to confirm the diagnosis. There were 423 cases in the OSAHS plus CAD group, and 774 cases in the OSAHS group.

Inclusion criteria were: (1) between the ages of 18 and 80; (2) overt CAD diagnosis per the “Guidelines for the Diagnosis and Treatment of Stable Coronary Heart Disease” (12); (3) clear diagnosis of OSAHS according to the 2017 “Clinical Practice Guideline for Diagnostic Testing for Adult Obstructive Sleep Apnea: An American Academy of Sleep Medicine Clinical Practice Guideline” (13).

Exclusion criteria were: (1) central sleep apnea; (2) concomitant severe cardiac, liver, renal insufficiency and malignant tumors; (3) concomitant chronic respiratory diseases, lung or other infections;(4) merge endocrine system diseases such as hyperthyroidism, hypothyroidism, and acromegaly; (5) concomitant primary systemic vasculitis; (6) concomitant mental illness, currently using sedative and hypnotic drugs. Each patient provided signed informed consent.

### Clinical data

The general information of the patients, including gender, age, medical history (such as history of hypertension and diabetes), family history, smoking history, and snoring status, was systematically recorded. Additionally, the neck circumference (in cm), height (in m²), and weight (in kg) of the patients were documented. The Body Mass Index (BMI) was calculated using the formula weight/height² (kg/m²). (2) Fasting blood sample collection was carried out the next morning following an 8 h fasting. Blood urea nitrogen (BUN), serum creatinine (Scr), triglycerides (TG), total cholesterol (TC), high-density lipoprotein cholesterol (HDL-C), low-density lipoprotein cholesterol (LDL-C), alanine aminotransferase (ALT), aspartate aminotransferase (AST) and fasting blood glucose (FBG) were measured using a Roche C8000 fully automated biochemical analyzer (Roche Diagnostics GmbH, Germany). (3) A Welch Allyn 6,100 dynamic blood pressure monitor was used to perform 24 h ambulatory blood pressure monitoring(blood pressure after medication). 24 h systolic (24 h SBP) and diastolic (24 h DBP) blood pressure levels were recorded upon completion of the monitoring.

### Coronary angiography (CAG)

Coronary angiography was performed by cardiovascular specialists. Preoperative procedures included routine electrocardiogram, blood pressure measurements, and oxygen monitoring. A GE Innova 2,100 digital subtraction angiography machine was used. The Seldinger puncture technique was applied, with a specific cardiac catheter introduced via the radial or femoral artery into the openings of the left and right coronary arteries. Contrast medium was injected, and selective left and right coronary angiography was conducted using the standard Judkins technique with many positions and angles. The results of the coronary angiography were analyzed by two or more cardiovascular disease specialists. Coronary artery lesions were examined for severity utilizing the Gensini score: Briefly, the score for each vessel stenosis degree was multiplied by the score for lesion location to obtain the lesion's score for each vessel. The total score for all lesions constituted the total coronary score for each patient, The higher the total score, the more severe the coronary artery disease (14).Record the number of coronary artery lesions, number of coronary vessels with ≥3 lesions indicating multivessel coronary disease.

### Coronary computed tomography angiography (CCTA)

Image acquisition was performed on a Siemens dual-source CT from Germany. The individuals were positioned supine for scanning from 1 cm below the tracheal carina to the level of the cardiac diaphragm. An enhanced scan employed a two-phase injection technique: during the initial step, 60–80 ml of non-ionic contrast agent was administered by injection into the median cubital vein at 3.5–5.0 ml/s; during the second step, 30 ml of physiological saline was administered as described for the contrast agent. The voltage and current were 120 kV and 380–410 mA, respectively. The data were imported into the workstation, and the corresponding volumetric data were assessed to obtain three-dimensional images of the coronary arteries and observe stenotic lesions. Diagnosis was independently completed by two experienced radiologists. The Gensini score was used to assess the CCTA data.

### Polysomnography (PSG)

A Compumedics polysomnography device manufactured in Australia was used to conduct a 7-h overnight PSG. Simultaneous monitoring included blood oxygen saturation, pulse, respiratory rate, snoring, and nasal airflow(All patients were not treated with Continuous Positive Airway Pressure,CPAP). Pre-monitoring education on precautions was provided to all patients. Post-monitoring, data analysis was performed using the Remlogic software, with the sleep report interpreted by a professionally trained physician and reviewed by a senior physician. Collected monitoring data included key indicators such as the apnea-hypopnea index (AHI), mean arterial oxygen saturation (MSaO_2_), and lowest arterial oxygen saturation (LSaO_2_).

### Screening scales

The 4 V includes gender (male, 4 pts; female, 0 pt), blood pressure level in mmHg (normal blood pressure, 1 pt; stage 1 hypertension, 2 pts; stage 2 hypertension, 3 pts; stage 3 hypertension, 4 pts), BMI in kg/m² (<21.0, 1 pt; 21.0–22.9, 2 pts; 23.0–24.9, 3 pts; 25.0–26.9, 4 pts; 27.0–29.9, 5 pts; ≥ 30.0, 6 pts) and self-reported snoring (almost daily or frequent snoring, 4 pts; others, 0 pt).

The BQ has a total of 10 questions, divided into snoring or apnea during sleep. Three categories are possible: fatigue or sleepiness in daily life and a history of hypertension or BMI ≥ 30 kg/m² (a “yes” answer to each question was worth 1 pt, and a “no” was attributed 0 pt).

The SBQ mainly involves snoring, daytime fatigue, apnea during sleep, hypertension, and BMI ≥35 kg/m², age ≥50 years old, neck circumference ≥40 cm, and male. A “yes” answer to each question was worth 1 pt, and a “no” was worth 0 pt. A total score for all screening scales was calculated. The personnel responsible for scoring have undergone standardized training.

### Statistical analysis

SPSS (26.0), R (4.3.2), OriginPro (2021) and GraphPad Prism (9.4.0) were employed for data analysis. The Shapiro–Wilk test was employed to test continuous variables for normality. Measurement data that conformed to normal distribution were reported as mean ± standard deviation ($\bar{x}\pm s$) and compared by the *t* test. Correlation analysis was carried out by *Pearson c*orrelation. Non-normally distributed measurement data were represented by M (P_25_, P_75_) and compared by non-parametric test, and *Spearman's* correlation analysis was used for correlation analysis. Count data were reported as frequency (%) and compared by the chi-square (*χ*^2^) test. LASSO regression analysis was employed to identify potential influencing factors of CAD in patients with OSAHS. To account for confounding variables, propensity score matching (PSM) was utilized. Bo/th univariate and multivariate logistic regression analyses were conducted on the adjusted dataset to determine independent risk factors for CAD in OSAHS patients. Subsequently, a nomogram model was developed based on these identified risk factors. The predictive performance of the model for CAD was evaluated using the ROC curve analysis, the Hosmer-Lemeshow test, calibration curves, and DCA. Additionally, the significance of each risk factor was assessed using the Random Forest machine learning algorithm, with model performance evaluated through Out-of-Bag (OOB) error derived from bootstrap sampling of unselected samples. A *P*-value of less than 0.05 was deemed statistically significant.

## Results

### General baseline data

Totally 1,197 OSAHS cases were examined in the current study, 423 of whom were accompanied by CAD and were 48.53 ± 9.38 years old. The OSAHS + CAD group had higher rate of males, hypertension, smoking history, and multivessel coronary disease, age, TG, AHI, Gensini scores, and 4 V, BQ, and SBQ vs. the OSAHS group. HDL-C, MSaO_2_ and LSaO_2_ were starkly lower in the OSAHS + CAD group vs. the OSAHS group (*P* < 0.05). Diabetes history, family history, BMI, BUN, Scr, TC, LDL-C, ALT, AST, FBG, 24 h SBP, and 24 h DBP were similar in both groups (*P* > 0.05, Table 1）.

**Table 1:** Comparison of baseline data between OSAHS group and OSAHS + CAD group.

Characteristics	OSAHS	OSAHS + CAD	*χ*2/Z/*t*	*P* ^b^
	(*n* = 774^a^)	(*n* = 423^a^)		
Gender[male/*n*(%)]	689 (89.0)	416 (98.3)	33.536	<0.001
Age(years)	(47.15 ± 9.39)	(51.06 ± 8.83)	−7.033	<0.001
Hypertension [yes/*n*(%)]	363 (46.9)	326 (77.1)	101.912	<0.001
Diabetes[yes/*n*(%)]	260 (33.6)	155 (36.6)	1.124	0.289
Smoking history[yes/*n*(%)]	340 (43.9)	221 (52.2)	7.600	0.006
Family history[yes/*n*(%)]	102 (13.2)	59 (13.9)	0.139	0.709
BMI(kg/m^2)^	25.95 (24.22,27.78)	27.68 (25.72,30.12)	−9.935	0.390
BUN(mmol/L)	5.3 (4.5,6.3)	5.5 (4.6,6.4)	−1.173	0.241
Scr(μmol/L)	70.05 (59.00,79.78)	71.80 (59.00,81.20)	−0.647	0.517
TG(mmol/L)	1.69 (1.15,2.51)	1.81 (1.28,2.72)	−2.337	0.019
TC(mmol/L)	4.27 (3.58,4.93)	4.33 (3.51,5.01)	−0.522	0.602
HDL-C(mmol/L)	1.03 (0.89,1.18)	0.96 (0.83,1.11)	−4.072	<0.001
LDL-C(mmol/L)	2.69 (2.14,3.22)	2.72 (2.09,3.31)	−0.548	0.584
ALT(U/L)	29.90 (20.20,33.12)	26.00 (19.59,33.30)	−1.963	0.050
AST(U/L)	24.70 (19.10,26.19)	23.92 (18.80,28.90)	−0.620	0.535
FBG(mmol/L)	5.41 (4.77,5.78)	5.30 (4.66,6.13)	−0.611	0.541
24 h SBP(mmHg)	131.0 (123.0,140.0)	133.0 (122.0,143.0)	−1.568	0.117
24 h DBP(mmHg)	85.0 (78.0,93.0)	86.0 (78.0,93.0)	−0.733	0.464
AHI(events/hour)	21.35 (13.70,28.35)	25.26 (16.70,39.90)	−6.394	<0.001
MSaO_2_(%)	92.5 (91.8,93.4)	92.4 (91.2,93.2)	−2.910	0.004
LSaO_2_(%)	82.0 (78.0,85.0)	81.0 (75.0,84.0)	−4.228	<0.001
Multivessel coronary disease[n(%)]	49 (6.3)	238 (56.3)	374.15	<0.001
Gensini scores(points)	0.0 (0.0,5.0)	19.0 (12.0,32.0)	−27.655	<0.001
The 4 V(points)	9.0 (8.0,11.0)	12.0 (10.0,14.0)	−17.370	<0.001
BQ(points)	1.0 (0.0,1.0)	1.0 (1.0,3.0)	−12.588	<0.001
SBQ(points)	2.0(2.0,3.0)	3.0(3.0,5.0)	−14.957	<0.001

^a^ *n*(%); M(P_25,_P_75_); ($\bar{x}\pm s$).

^b^ Pearson's Chi-squared test;Wilcoxon rank sum test;*T*-test.

### LASSO regression analysis

**Figure 1:**
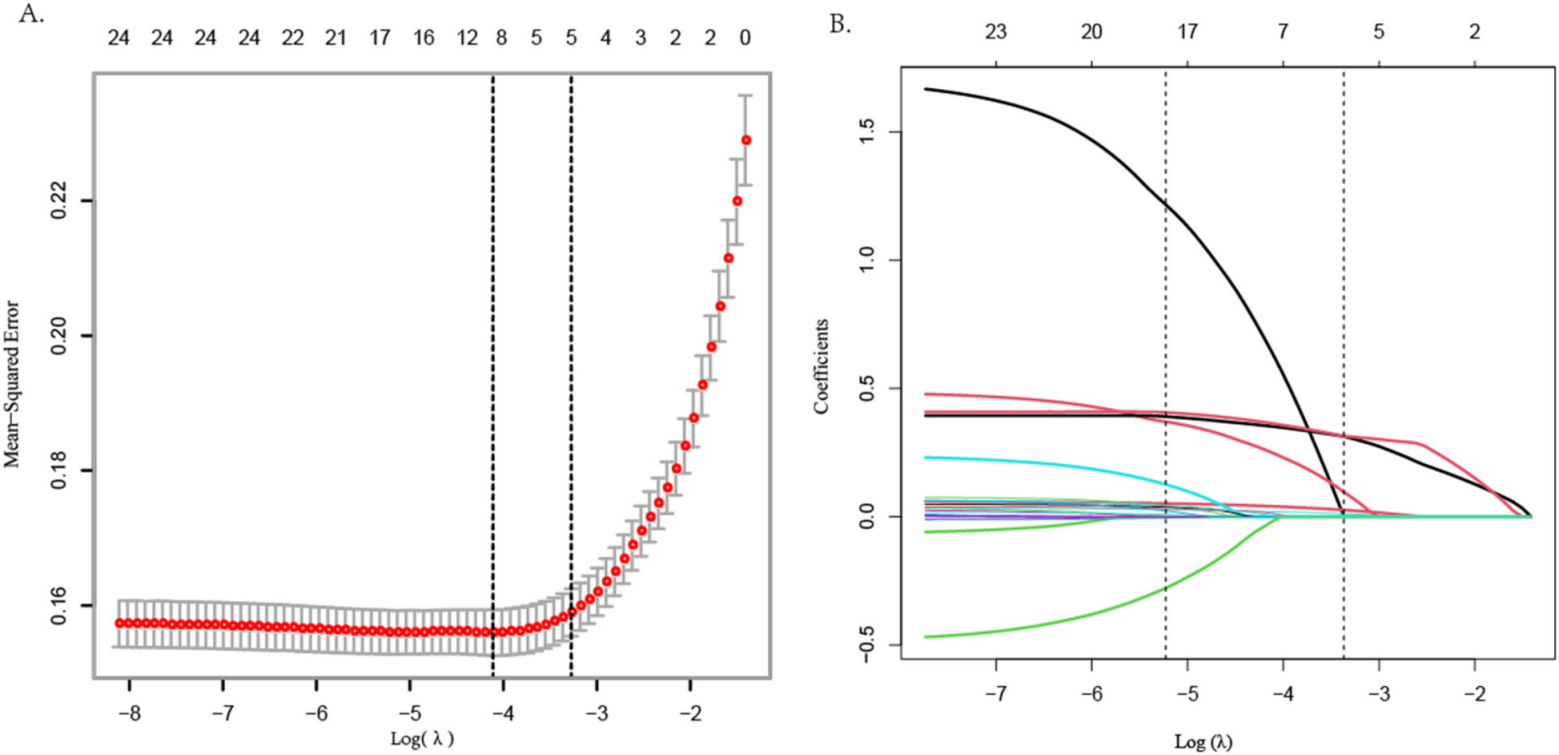
LASSO regression variable screening.

LASSO regression was employed to screen CAD risk factors. The tuning parameter (*λ*) was chosen by 10-fold cross-validation with the minimum criterion, and a dashed line was drawn at the optimal value determined based on the minimum criterion and 1 standard error of the minimum criterion (*λ*1se) (Figure 1A). A LASSO coefficient distribution map of 24 texture features was obtained, and the coefficient profile was generated based on the log (*λ*) sequence (Figure 1B). When *λ*1se = 0.034, 5 non-zero coefficient predictors were screened out (*P* < 0.05), namely age, hypertension, AHI, 4 V, and SBQ, which were potential risk factors for CAD.

### Propensity score matching analysis

Propensity Score Matching (PSM) analysis was conducted on the irrelevant factors identified through LASSO regression analysis(including gender, history of diabetes, smoking history, family history, BMI, BUN, Scr, TG, TC, HDL-C, LDL-C, ALT, AST, FBG, 24 h SBP, 24 h DBP,MSaO_2_, LSaO_2_, BQ). The analysis utilized a caliper value of 0.02 and a 1:1 matching ratio to mitigate the potential influence of these confounding variables on the study outcomes. The results of the PSM analysis indicated that there were no statistically significant differences between the two patient groups regarding history of diabetes, family history, BMI, BUN, Scr, TC, LDL-C, AST, FBG, 24 h SBP, and 24 h DBP (*P* > 0.05). Furthermore, following the PSM analysis, no significant statistical differences were observed between the two groups concerning gender, smoking history, TG, HDL-C, MSaO_2_, LSaO_2_, and BQ (*P* > 0.05) (see Table 2).

**Table 2:** Comparison of baseline data of OSAHS patients after PSM analysis.

Characteristics	OSAHS	OSAHS + CAD	$\chi^{2}$/Z/t	*P* ^b^
	(*n* = 293^a^)	(*n* = 293^a^)		
Gender[male/*n*(%)]	290 (99.0)	287 (98.0)	0.451	0.502
Age(years)	47.0 (40.0,52.0)	51.0 (46.0,56.0)	−6.238	<0.001
Hypertension [yes/*n*(%)]	161 (54.9)	224 (76.5)	30.055	<0.001
Diabetes[yes/n(%)]	104 (35.5)	106 (36.2)	0.030	0.863
Smoking history[yes/*n*(%)]	146 (49.8)	146 (49.8)	0.000	1.000
Family history[yes/*n*(%)]	38 (13.0)	42 (14.3)	0.232	0.630
BMI(kg/m^2)^	27.16 (25.06,28.93)	26.49 (25.13,28.38)	−1.267	0.205
BUN(mmol/L)	5.50 (4.60,6.50)	5.40 (4.60,6.43)	−0.106	0.916
Scr(μmol/L)	71.00 (60.45,79.05)	70.58 (59.50,81.52)	−0.097	0.923
TG(mmol/L)	1.72 (1.16,2.63)	1.69 (1.20,2.62)	−0.117	0.907
TC(mmol/L)	4.23 (3.58,4.86)	4.32 (3.47,5.02)	−0.590	0.555
HDL-C(mmol/L)	0.99 (0.86,1.13)	0.99 (0.86,1.15)	−0.174	0.862
LDL-C(mmol/L)	(2.75 ± 0.81)	(2.74 ± 0.86)	2.968	0.085
ALT(U/L)	29.23 (20.55,33.42)	26.00 (18.90,33.49)	−1.988	0.047
AST(U/L)	24.70 (19.40,26.48)	23.20 (18.70,28.05)	−0.950	0.342
FBG(mmol/L)	5.42 (4.72,5.7)	5.30 (4.66,6.14)	−0.309	0.758
24 h SBP(mmHg)	131.0 (123.0,141.0)	133.0 (122.0,142.0)	−0.067	0.946
24 h DBP(mmHg)	(85.55 ± 11.04)	(85.67 ± 10.71)	0.669	0.410
AHI(events/hour)	22.23 (15.70,31.45)	23.38 (16.03,36.80)	−1.629	0.103
MSaO_2_(%)	92.40 (91.70,93.05)	92.50 (91.50,93.25)	−0.335	0.738
LSaO_2_(%)	81.00 (76.50,84.50)	81.00 (77.00,85.00)	−0.601	0.548
Multivessel coronary disease[n(%)]	18 (6.1)	156 (53.2)	155.672	<0.001
Gensini scores(points)	0.0 (0.0,5.0)	18.0 (12.0,31.0)	−19.662	<0.001
The 4 V(points)	10.0 (9.0,12.0)	11.0 (10.0,12.5)	−5.877	<0.001
BQ(points)	1.0 (1.0,2.0)	1.0 (1.0,2.0)	−0.552	0.581
SBQ(points)	3.0(2.0,3.0)	3.0(2.0,4.0)	−4.340	<0.001

^a^ *n*(%); M(P_25,_P_75_); ($\bar{x}\pm s$).

^b^ Pearson's Chi-squared test; Wilcoxon rank sum test; *T*-test.

### Univariable and multivariable analyses

**Figure 2:**
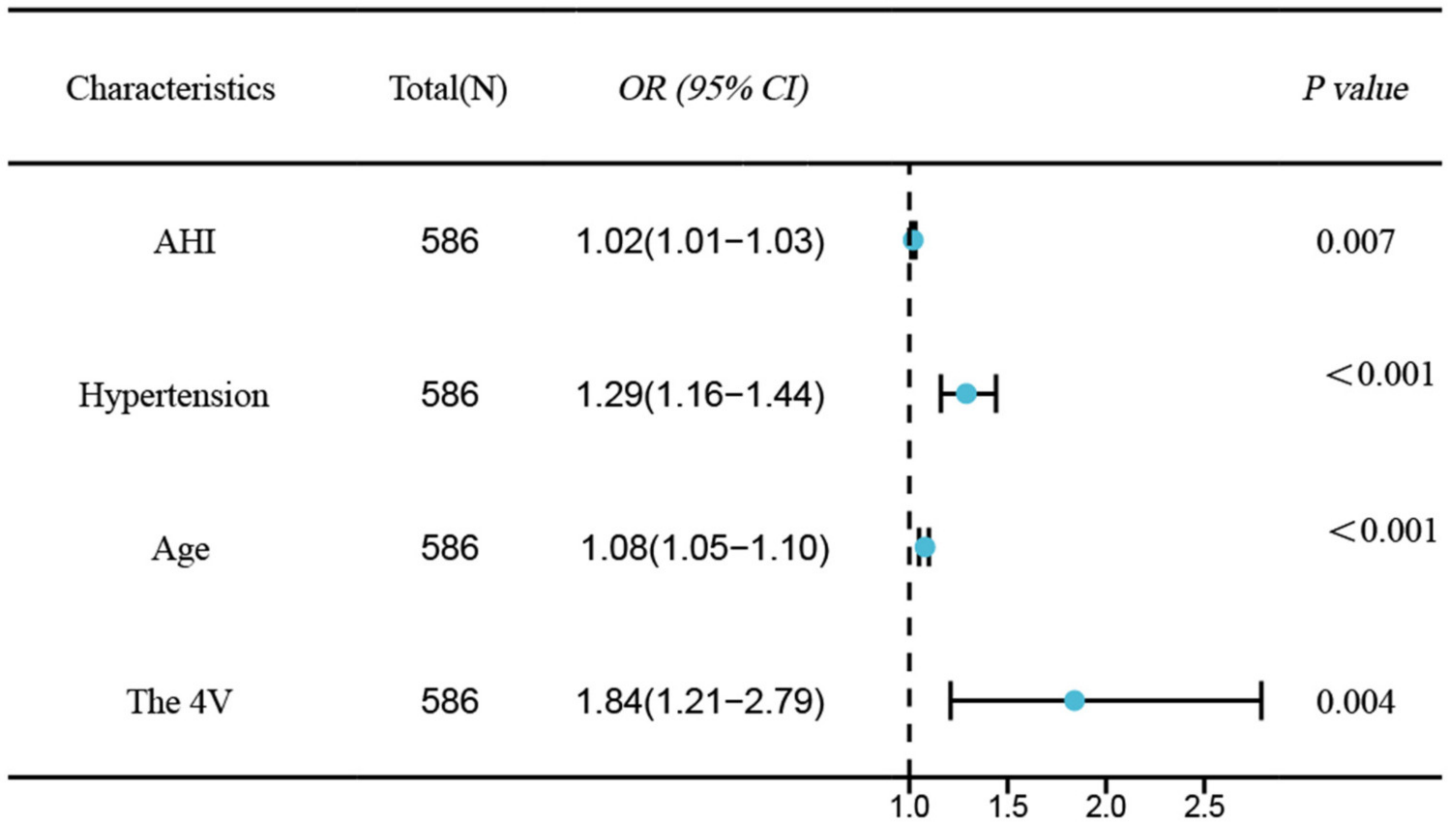
Forest plot of independent risk factors for CAD in OSAHS.

Univariable logistic regression analysis revealed age, hypertension, AHI, 4 V, and SBQ were significantly related to CAD occurrence in OSAHS cases. Employing the above indexes as independent variates and CAD as the dependent variate(1 = yes, 0 = no), binary logistic stepwise-regression analysis demonstrated age (*OR*=1.07, 95% CI: 1.05–1.1, *P* < 0.001), hypertension (*OR*=1.29, 95% CI: 1.16–1.44, *P* < 0.001), AHI (*OR*=1.02, 95% CI: 1.01–1.03, *P* = 0.007), and 4 V(*OR*=1.84, 95% CI: 1.21–2.79, *P* = 0.004)independently predicted OSAHS combined with CAD (Table 3 and Figure 2).

**Table 3:** Univariate and multivariate logistic regression analysis.

Characteristics	Univariate logistic regression			Multivariate logistic regression		
	*OR*	95% *CI*	*P*	*OR*	95% *CI*	*P*
Gender(male)	0.49	0.12–2.00	0.323	-	-	-
Age(years)	1.06	1.04–1.08	<0.001	1.07	1.05–1.10	<0.001
Hypertension(yes)	2.66	1.87–3.80	<0.001	1.29	1.16–1.44	<0.001
Diabetes(yes)	1.03	0.74–1.44	0.863	-	-	-
Smoking history(yes)	1.07	0.78–1.48	0.681	-	-	-
Family history(yes)	1.03	0.65–1.64	0.906	-	-	-
BMI(kg/m^2^)	0.97	0.92–1.04	0.420	-	-	-
BUN(mmol/L)	0.96	0.86–1.07	0.459	-	-	-
Scr(μmol/L)	1.00	0.99–1.01	0.883	-	-	-
TG(mmol/L)	1.00	0.90–1.11	0.977	-	-	-
TC(mmol/L)	1.06	0.91–1.24	0.435	-	-	-
HDL-C(mmol/L)	1.16	0.63–2.12	0.636	-	-	-
LDL-C(mmol/L)	0.99	0.82–1.20	0.921	-	-	-
ALT(U/L)	0.99	0.98–1.01	0.438	-	-	-
AST(U/L)	1.00	0.98–1.02	0.693	-	-	-
FBG(mmol/L)	1.02	0.92–1.13	0.687	-	-	-
24 hSBP(mmHg)	1.00	0.99–1.01	0.972	-	-	-
24 hDBP(mmHg)	1.00	0.99–1.02	0.891	-	-	-
AHI(events/hour)	1.02	1.00–1.03	0.006	1.02	1.01–1.03	0.007
MSaO_2_(%)	1.00	0.91–1.11	0.940	-	-	-
LSaO_2_(%)	1.00	0.97–1.02	0.788	-	-	-
The 4 V(points)	1.29	1.18–1.41	<0.001	1.84	1.21–2.79	0.004
BQ(points)	1.08	0.87–1.34	0.479	-	-	-
SBQ(points)	1.45	1.23–1.70	<0.001	-	-	-

### Constructing a nomogram model for patients with OSAHS combined with CAD

**Figure 3:**
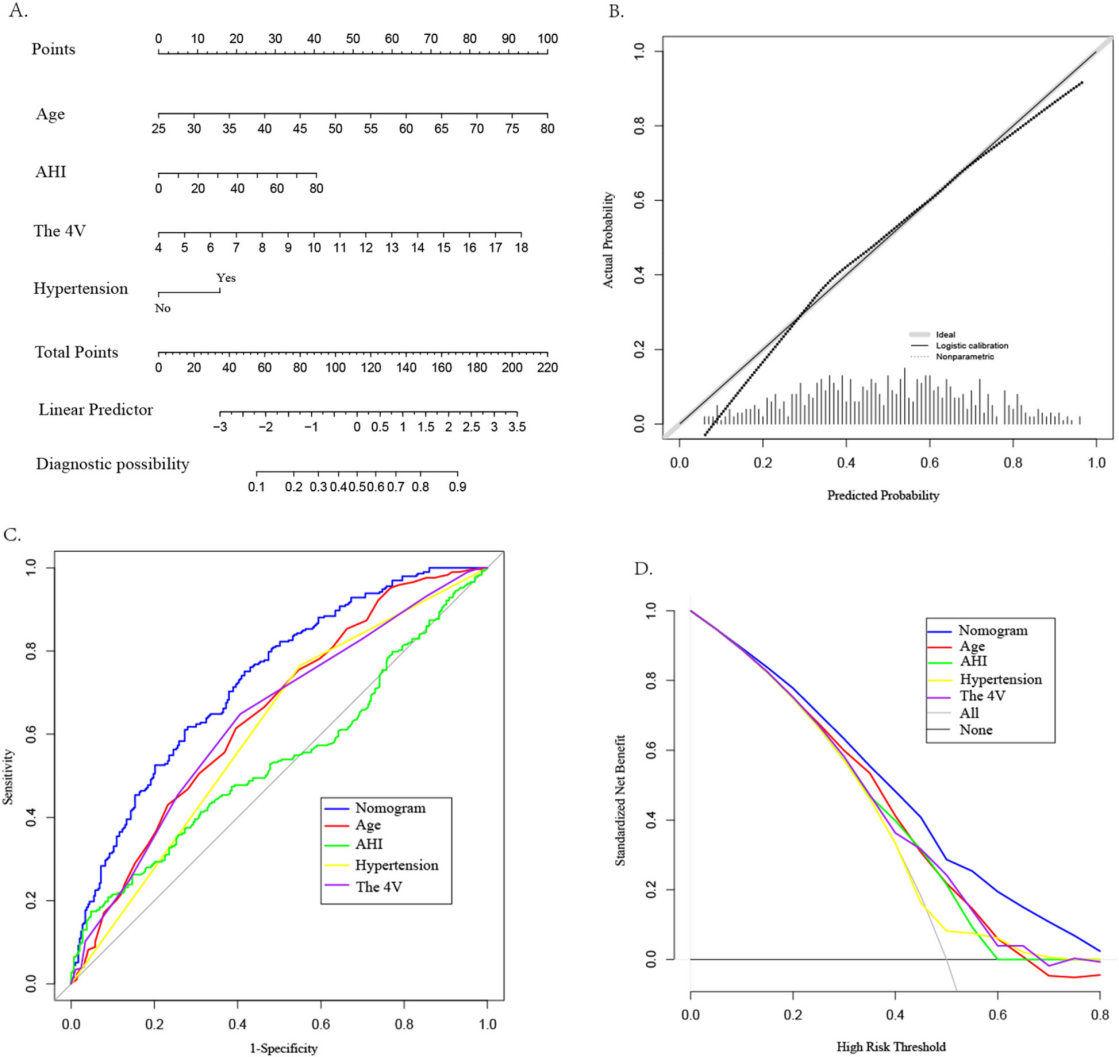
Construction of CAD nomogram **(A)**, calibration curve **(B)**, ROC curve **(C)**, and DCA **(D)** for OSAHS patients.

This study employed both univariate and multivariate logistic regression analyses to identify factors influencing CAD in patients with OSAHS. The analysis revealed four significant factors with predictive value for CAD. A nomogram model was constructed based on these four factors (see Figure 3A). The model was subjected to Hosmer-Lemeshow testing and calibration curve plotting, demonstrating that the actual risk of CAD occurrence in OSAHS patients closely aligns with the predicted risk, indicating a high level of consistency between the two (*χ*² = 6.39, *P* = 0.700; *P* > 0.05) (see Figure 3B). Furthermore, this study utilized the AUC value to evaluate the predictive efficacy of various factors in screening OSAHS patients for CAD. The results indicated that age (AUC = 0.649), hypertension (AUC = 0.608), AHI(AUC = 0.539), and the 4 V(AUC = 0.638) demonstrated varying degrees of predictive accuracy. When these four predictive factors were assessed in combination, their predictive accuracy significantly improved (AUC:0.729; 95% *CI*: 0.689–0.770; *P* < 0.05), yielding a sensitivity of 61.8%, specificity of 72.0%, positive predictive value of 68.8%, and negative predictive value of 65.3%(see Figure 3C). The model's discriminative ability surpassed that of each individual factor. Furthermore DCA results indicated that the predictive curves of all factors were predominantly above the'no intervention' and “complete intervention” curves, with the 4 V model providing a greater net benefit at the same disease probability (see Figure 3D).

### Evaluation accuracy of the machine learning model

**Figure 4:**
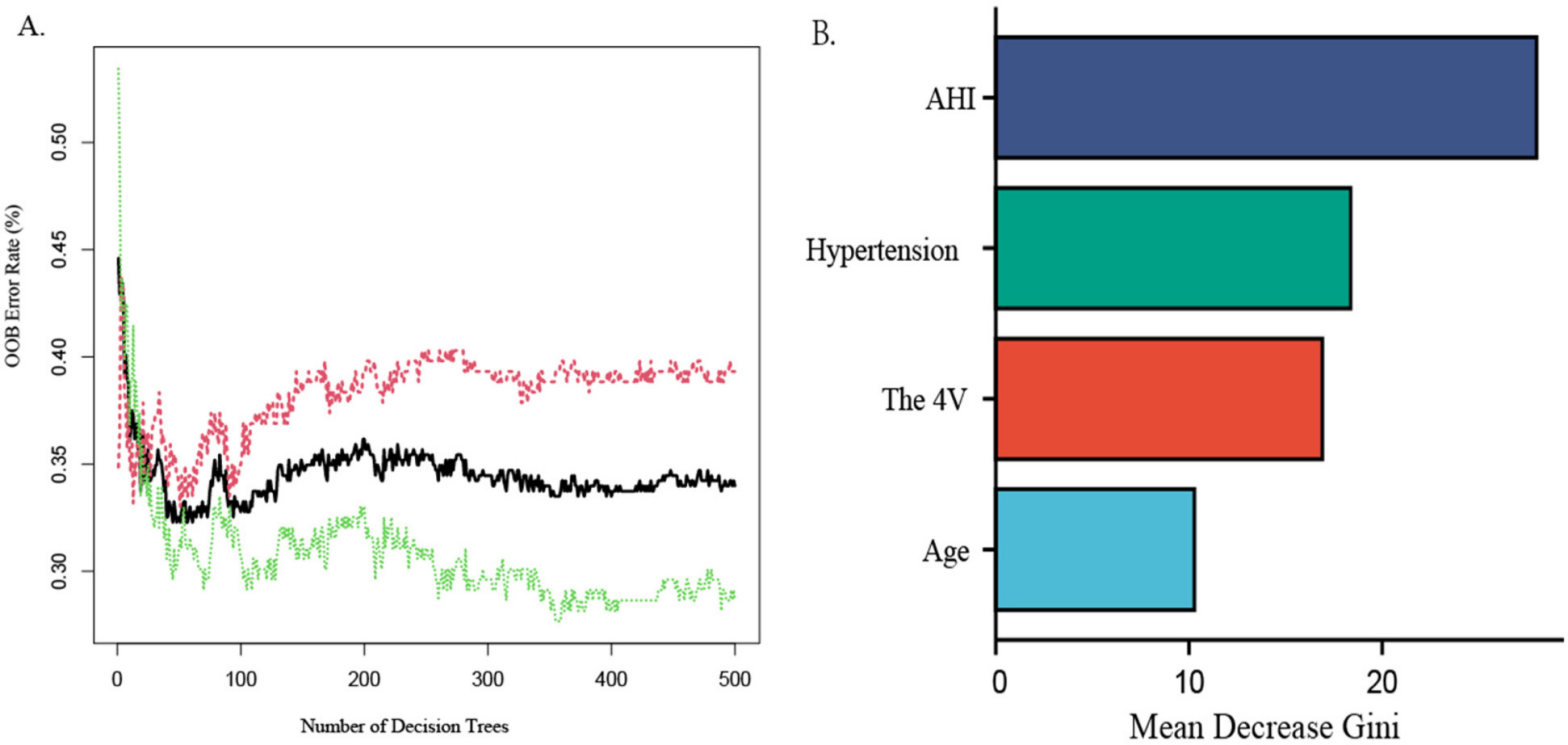
Random forest graph out-of-Bag error graph **(A)**, variable importance graph **(B)**

The random forest machine learning algorithm was utilized to further validate the model's accuracy in predicting the risk of CAD in patients with OSAHS and to evaluate the predictive significance of the four identified predictors. The results indicated that the random forest model achieved an AUC value of 0.729 (95% *CI*:0.562–0.709; *P* < 0.05) on the training set, demonstrating a sensitivity of 60.9%, specificity of 66.7%, positive predictive value of 64.6%, and negative predictive value of 63.0%. Utilizing an estimated 500 decision trees, with 2 variables at each node, the Out-of-Bag (OOB) mean squared error was calculated to be 33.98%. Notably, when the number of decision trees exceeded 300, the OOB error stabilized, indicating that further increases in the number of decision trees did not result in overfitting, with strong generalization ability[refer to Figure 4A, *Y*-axis label:OOB Error Rate (%), *X*-axis label:Number of Decision Trees]. Furthermore, the importance ranking of predictors for OSAHS combined with CAD was identified as follows: AHI, hypertension, the 4 V, and age (refer to Figure 4B).

### Associations among various variables and between predictors and Gensini scores

**Figure 5:**
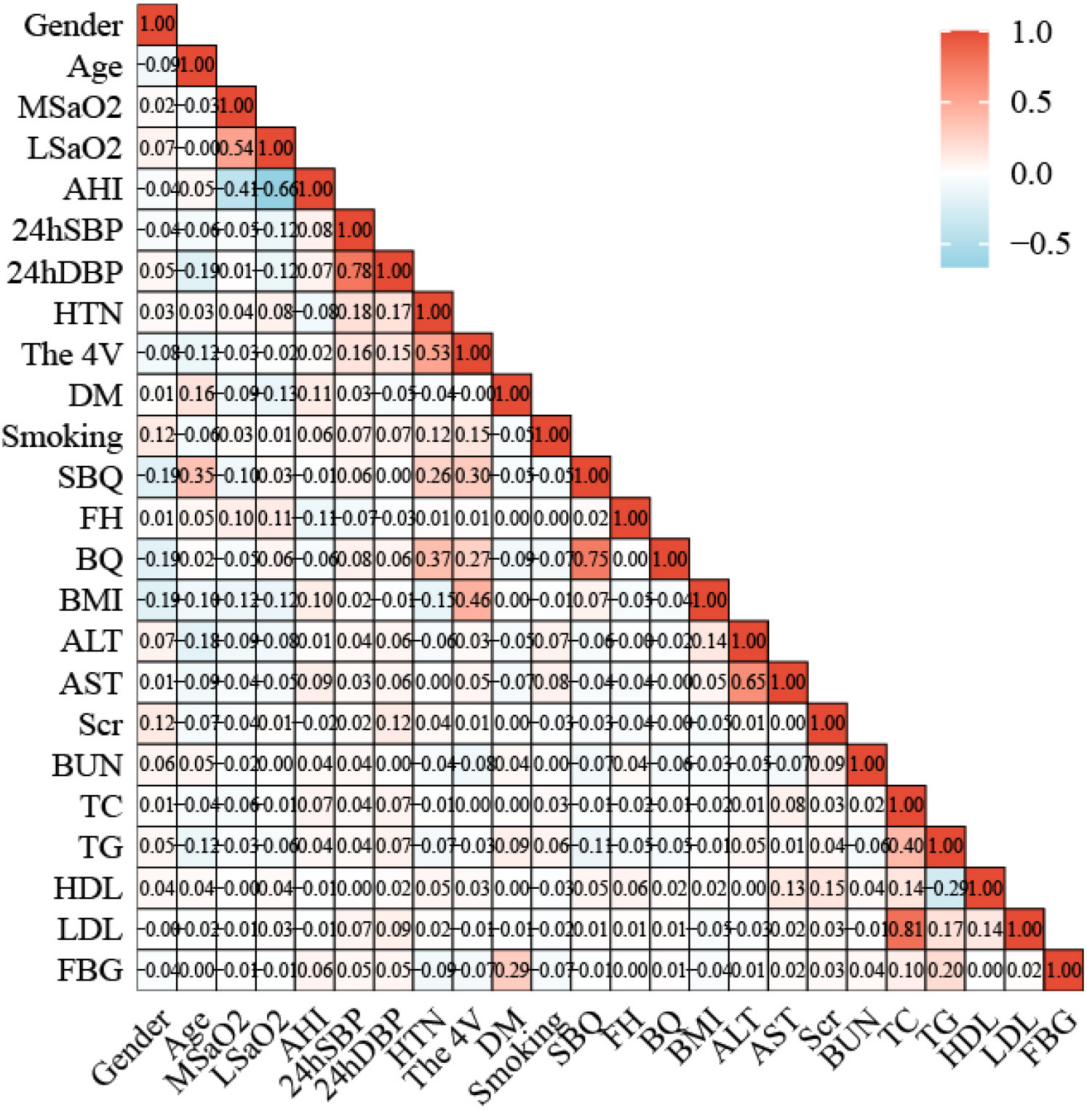
Correlation heat map between 24 variables.

**Figure 6:**
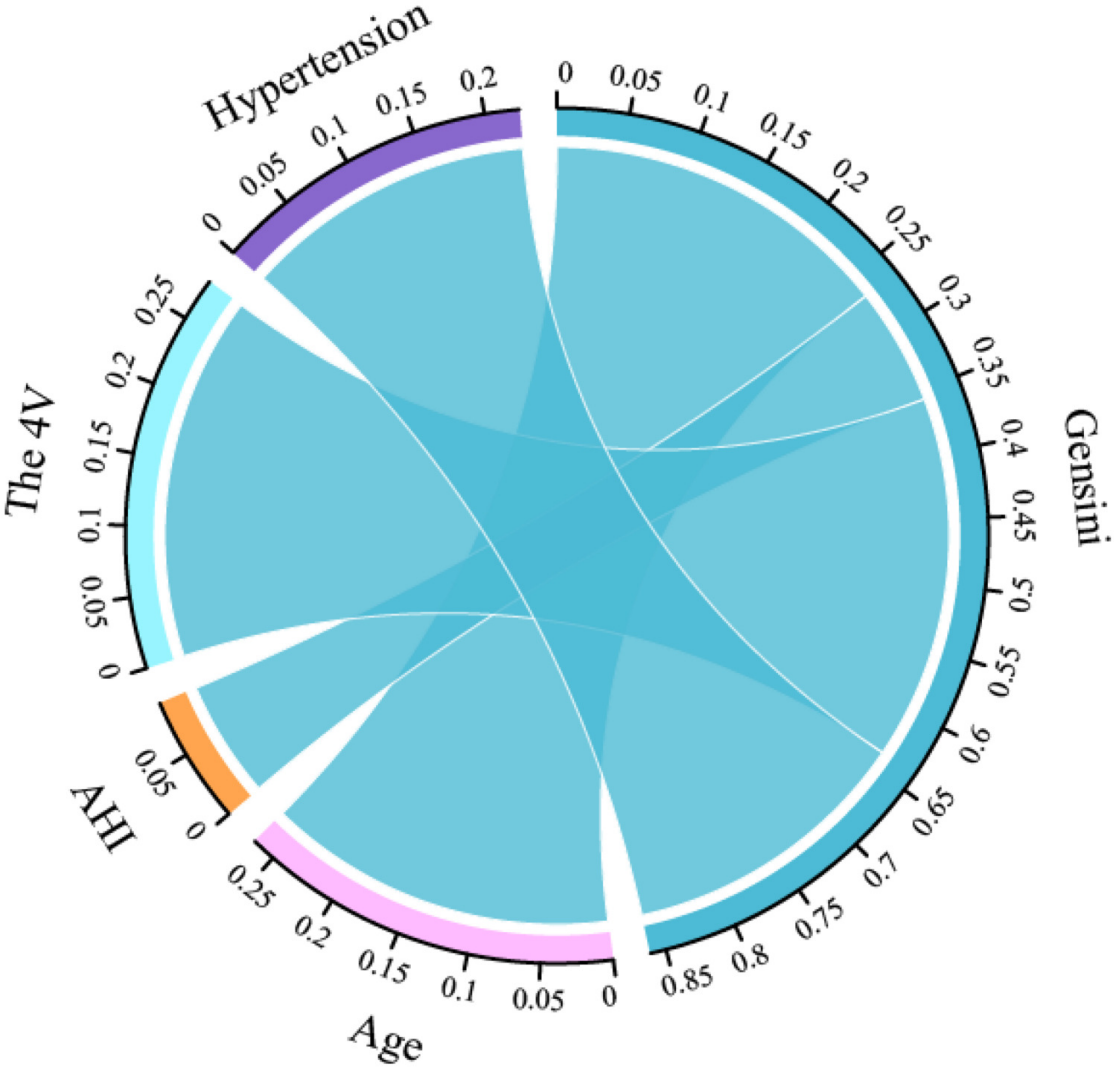
Correlation chord diagram between predictors and gensini scores.

The *Pearson's* or *Spearman's* correlation coefficient was employed to assess the associations among all variables (Figure 5). In the correlation matrix, there was a linear relationship between 4 V and hypertension (0.53), a linear relationship between AHI and LSaO_2_ (−0.66), and a linear relationship between BQ and SBQ (0.75). *Spearman's* correlation analysis was conducted to test the relationships between predictors and Gensini scores, and the results showed that age r = 0.267, *P* < 0.05), AHI (r = 0.090, *P* < 0.05), hypertension ((r = 0.224, *P* < 0.05) and 4 V (r = 0.280, *P* < 0.05) had significant correlations with Gensini scorse (Figure 6).

## Discussion

OSAHS is closely related to various diseases such as CAD, hypertension, diabetes, and stroke. The pathophysiological mechanisms of OSAHS are currently unclear, but might be related to overactive sympathetic nervous system, induced inflammation, oxidative stress and vascular endothelial dysfunction. Insulin resistance is associated with dysregulated lipid metabolism, which might further elevate cardiovascular risk (15, 16). However, in actual clinical setting, the diagnosis rate of OSAHS is not high. Intermittent hypoxia occurring at night in patients with OSAHS may trigger angina and even lead to acute myocardial infarction. In previous studies, only a small number of patients underwent comprehensive polysomnography, which suggests that in many CAD patients, concurrent OSAHS may be missed. Therefore, the identification and screening of OSAHS combined with CAD should be improved in clinical practice (11).

Currently, a variety of scales are available for OSAHS screening, but have limitations such as numerous items and insufficient accuracy, and some contents are not suitable for the actual situation of the Chinese population. The 4 V only includes four indexes, i.e., gender, blood pressure, BMI, and snoring. It showed high sensitivity and specificity in a previous study and significantly reduced the false negative and false positive rates when screening for severe OSAHS, thereby improving the accuracy of the scale (17). BQ is a commonly used tool for screening OSAHS. Although its structure is relatively complex, it has undergone extensive validation studies and has high sensitivity. The SBQ was expanded by Chung et al. based on the STOP questionnaire. Silva et al. (4) used the Sleep Heart Health Study Population (SHHS) database to conduct a retrospective analysis of the data of 4,770 subjects, evaluating the ESS, 4 V, STOP questionnaire and the predictive performance of SBQ. The results demonstrated sensitivities for the STOP questionnaire in predicting moderate and severe OSAHS of 62% and 68.8%, respectively, vs. 87% and 70.4% for the SBQ, respectively. A cross-sectional study by Koseoglu and collaborators (18) explored the relationship between the NoSAS score and cardiovascular disease in OSAHS patients. The results revealed the screening questionnaire was associated with cardiovascular disease severity, while the 4 V, BQ and SBQ had multiple variables, consistent with the NoSAS score, and may also be tightly associated with CAD occurrence and development.

The baseline data after PSM in this study showed that the OSAHS + CAD group had elevated age and hypertension rate (*P* < 0.05). OSAHS is often accompanied by the above traditional risk factors for CAD and can directly induce atherosclerosis. A series of intermediate mechanisms further cause various cardiovascular diseases including CAD (19). Meanwhile, AHI, the proportion of multivessel coronary disease, and Gensini score increased significantly, all indicating that the relationship between OSAHS and CAD is intricate; in addition, OSAHS can aggravate the severity of coronary atherosclerosis, which is consistent with most previous studies. Research (20, 21) is consistent with the 4 V and SBQ being markedly elevated in the OSAHS + CAD group vs. the OSAHS group. The correlation analysis also demonstrated the Gensini scores had positive correlations with 4 V scores. This may be because CAD patients basically have problems in these two scales. It is related to factors such as male gender, overweight and high blood pressure. These factors can aggravate the degree of coronary atherosclerosis. The difference in BQ score between the groups had no statistical significance. This may be because 4 V and SBQ scores are richer in content, and the scoring items are divided in more detail. It may also be because anthropometric characteristics, including BMI and neck circumference, are involved in OSAHS and CAD as common risk factors (22).

Multivariate logistic regression analysis in this study found that age, hypertension, AHI, and 4 V were independent risk factors for CAD in OSAHS cases and tightly associated with CAD prediction and the severity of coronary atherosclerotic lesions. Among them, age and hypertension are traditional risk factors for CAD, as verified in a large number of studies such as Framingham (23–25).The pathophysiological mechanisms underlying the link between hypertension and CAD are complex and include overactivation of neurohormones, accelerated development of the atherosclerotic plaque, endothelial dysfunction, altered intramyocardial coronary circulation (26).A study investigated the effects of adherence to CPAP therapy (>4 h/night) and demonstrated that, compared to OSAHS patients assigned to the usual care group, patients who adhered to CPAP had significantly lower mean office blood pressure, systolic blood pressure, and diastolic blood pressure (27). None of the patients in this study used CPAP. The LASSO regression selected 5 predictors (including SBQ), yet SBQ was excluded in the final multivariate model, SBQ was significant in LASSO regression and univariate logistic regression analysis, but was excluded in multivariate analysis, indicating that its predictive power might be overshadowed by other stronger or more direct variables. The current study further built a clinical prediction nomogram model for CAD in OSAHS cases. ROC curve analysis showed that age (AUC = 0.649), hypertension (AUC = 0.608), AHI(AUC = 0.539), and the 4 V(AUC = 0.638) had basically the same predictive ability for the occurrence of CAD, which might be associated with the sample sizes of the included studies. Meanwhile, the joint application of the 4 independent risk factors had better predictive ability for CAD (AUC: 0.729; 95% *CI*: 0.562–0.709; *P* < 0.05), which is relatively high. The above AUC value shows that this model can well identify OSAHS cases complicated by CAD, which may help further understand the basic roles of these four variables in assessing the risk of OSAHS complicated by CAD. Through the Hosmer-Lemeshow test and DCA, it was found the model has good clinical robustness in CAD prediction in OSAHS cases and is practical in clinical risk stratification. Our prediction model uses the screening scale as a risk factor and emphasizes the association of OSAHS screening scale with CAD. Because of limited traditional risk factors for CAD, it is currently challenging to further develop predictive models in CAD (28).

This was further confirmed in the random forest graph machine model, and the predictive value of AHI was the highest. When using OOB error to evaluate model performance with the samples not selected by Bootstrap, the OOB error of 33.98% indicates an accuracy rate of 66.02%, suggesting that the model has certain predictive capabilities. The random forest graph machine learning algorithm is a collection of decision trees used to train the algorithm, representing a collection of multiple decision trees to minimize overfitting. A retrospective study of 967 adults found that after comparing six machine learning models generated through clinical indexes obtained via stepwise variable selection, the potential of the built model in predicting cardiovascular disease risk in patients with OSAHS was calculated. The sensitivity of the random forest graph machine model was 84% and the specificity was 99%, suggesting good predictive performance (10). AHI is an indicator for judging the severity of OSAHS. Severe OSAHS cases have a significantly increased risk of CAD, which is consistent with research findings by Ishiwata et al. (29) and Pei et al. (30). Intermittent hypoxia and reoxygenation at night in patients with severe OSAHS can induce oxidative stress, initiate an inflammatory cascade, increase sympathetic nerve activity, lead to endothelial dysfunction, suppress metabolic regulation, promote platelet aggregation, and trigger a series of pathophysiological changes, thereby promoting the occurrence and development of atherosclerosis, ultimately leading to an increased risk of CAD (31).

The innovation of this study is that it is the first to link the 4 V, BQ and SBQ with CAD, analyze the associations of 4 V with the severity of coronary atherosclerosis, and incorporate it into the predictive model. The research findings show that the model has high clinical application value. Our results have important implications for the risk stratification of OSAHS patients, providing support for healthcare professionals to adopt a multifaceted approach in the assessment and management of patients with OSAHS, potentially improving patient outcomes and reducing the occurrence of cardiovascular events.

## Limitations

The present study still had limitations. First, it was performed at a single center, with all samples obtained from the same hospital, which may limit the applicability of the results to a wider population. The findings should be cautiously validated when applied to other regions or primary care settings. Additionally, a retrospective design may introduce bias related to selection and data collection. For example, reliance on self-reported questionnaires may lead to inaccuracies in assessing sleep apnea severity due to potential recall bias or subjective interpretation. Finally, although this study used PSM to balance potential confounders, residual confounding could not be completely ruled out. Further studies are required to replicate the current findings in different cohorts and to examine the longitudinal impacts of these screening scales on cardiovascular outcomes in patients with OSAHS. Additionally, extending research to biomarkers or imaging techniques may provide sound insights into the pathophysiological mechanisms of OSAHS and coronary artery disease.

## Conclusions

In summary, Age, hypertension, AHI, and the 4 V are independent risk factors for CAD in patients with OSAHS. The 4 V scores were markedly elevated in individuals with OSAHS combined with CAD and showed positive correlations with the severity of coronary atherosclerosis. We also built a nomogram model for predicting CAD occurrence by incorporating the OSAHS screening scale into the diagnostic system. Because of its simplicity, convenience, non-invasiveness and other characteristics, the model has great application value in clinical screening. It is expected to become a new tool for screening OSAHS combined with CAD and has certain application value in clinical practice. The developed model may help identify OSAHS cases with a high risk of cardiovascular disease. However, its clinical diagnostic value needs further investigation.

## Data Availability

The original contributions presented in the study are included in the article/Supplementary Material, further inquiries can be directed to the corresponding author.
